# Long non-coding RNA CRNDE exacerbates NPC advancement mediated by the miR-545-5p/CCND2 axis

**DOI:** 10.1186/s12935-021-02348-2

**Published:** 2021-12-04

**Authors:** Sichen Ge, Chengyi Jiang, Min Li, Zhongqiang Cheng, Xiaojia Feng

**Affiliations:** 1 Department of Otorhinolaryngology Head and Neck Surgery, The First Affiliated Hospital of Bengbu Medical College, Bengbu, 233000 Anhui China; 2 Department of Surgical Oncology, The First Affiliated Hospital of Bengbu Medical College, Bengbu, 233000 Anhui China

**Keywords:** LncRNA CRNDE, NPC, miR-545-5p, CCND2

## Abstract

**Background:**

Previous studies indicated CRNDE to have a pivotal part within tumorigenesis. Notwithstanding, precise details on CRNDE activities within NPC are still uncertain. The investigation described in this article served to focus in greater depth on the mechanistics regarding CRNDE, together with all associated regulatory networks, on nasopharyngeal carcinoma (NPC) and its treatment possibilities.

**Methods:**

Quantitative real-time polymerase chain reaction (RT-qPCR) analyzed CRNDE, miR-545-5p and CCND2 expression within NPCs and representative cell lineages. CCK-8 cell counting-, EdU-, wound-healing-/transwell-assays analyzed cellular proliferation, migrative, together with invasive properties. Apoptosis/cell cycle progression were scrutinized through flow cytometry. Dual-luciferase reporter assays validated CRNDE/miR-545-5p/CCND2 interplay. Proteomic expression of apoptosis-related protein, EMT-related protein and CCND2 protein were evaluated through Western blotting. In addition, Ki67 expression was evaluated through immunohistochemical staining. The effect of CRNDE in vivo was assessed by nude murine xenograft model studies.

**Results:**

This study demonstrated up-regulated expression of CRNDE and CCND2 within NPC tissues/cell lines. Meanwhile, miR-545-5p was down-regulated. CRNDE knock-down or miR-545-5p over-expression drastically reduced NPC proliferative, migrative and invasive properties, promoted apoptosis/altered cell cycle, and inhibited CCND2 expression. However, miR-545-5p down-regulation had opposing effects. All inhibiting functions generated by CRNDE down-regulation upon NPC progression could be counterbalanced or synergistically exacerbated, depending on miR-545-5p down-regulation or up-regulation, respectively. Multiple-level investigations revealed CRNDE to serve as a sponge for miR-545-5p, and can target CCND2 within NPCs.

**Conclusions:**

CRNDE increases CCND2 expression by competitive binding with miR-545-5p, thus accelerating the development of NPC. This provides potential therapeutic targets and prognostic markers against NPC.

## Background

Nasopharyngeal carcinoma (NPC) is an epithelial malignant tumor, commonly manifested within the pharyngeal recess and is distinctly prevalent within the regions of East and Southeast Asia [1]. Radio-and chemo-therapy form the main therapies. Most patients having early symptoms are challenging to diagnose, and consequently, the disease develops rapidly [2]. The survival rate within NPC patients is typically low [1], consequently emphasizing the importance of identifying and developing more effective NPC therapeutic measures. Therefore, more detailed pathogenesis of NPC and advanced treatment methods must be unraveled.

Long non-coding RNAs (lncRNAs) are > 200 bp and lack an open reading frame that cannot encode proteins [3]. Due to their vast spectrum of expression profiles and tissue-linked expression specificity, lncRNAs can be used as tumor markers and therapeutic targets [4]. Dysregulated expression/mutations in lncRNAs influences tumorigenesis and metastasis, with this trend occurring within multiple tumor models including colon [5], prostate [6] and oral cancer [7]. Colorectal neoplasia differential expression (CRNDE) is a long noncoding RNA with high-sensitivity/-specificity within plasma and tumor tissues. CRNDE was found to be implicated within multiple tumor processes [8]. Apart from colorectal cancer, CRNDE is also up-regulated in hepatic cancer [9], cervical cancer [10] and clear-cell-renal-cell carcinoma [11], promoting tumor expansion, invasiveness and metastases. In some head and neck tumors, CRNDE also plays a role in promoting cancer. For example, studies have found that CRNDE plays an oncogenic role in the development of TSCC by inhibiting the expression of miR-384 [12]. However, the mechanism/s employed by CRNDE to influence NPC malignancy is lacking.

MicroRNAs (miRNAs) entail small non-coding RNAs (20–22 nucleotides long), that possess pivotal regulatory functions upon physiological/developmental processes, within all cell-types [13]. MiRNA dysregulation is linked to a vast spectrum of human conditions, such as tumors. Previous literature demonstrated miR-545-5p to be down-regulated within colon adenocarcinoma and contributes to its proliferative, apoptotic, migrative and invasive properties [14]. However, previous literature reports regarding the influence of miR-545-5p over NPC tissue phenotypic characteristics are still scarce. In addition, cyclin D2 (CCND2), as a member of the highly-conserved cyclin family, has protein-independent periodicity throughout the cell cycle [15] and is closely linked to NPC manifestation and development [16]. However, no previous reports exist for confirming miR-545-5p to regulate CCND2.

LncRNAs have microRNA Responsible Elements (MRE), a sponge-binding site for miRNAs, in order to regulate specific miRNA-orchestrated target transcript down-regulation [17]. Chen and colleagues highlighted lncRNA CRNDE to promote angiogenesis within hepatoblastoma, through targeted activity on the miR-203/VEGFA axis. Zhu et al. confirmed that CRNDE promotes proliferative/angiogenesis properties by pancreatic cancer, through regulation of miR-451a and CDKN2D [18]. Based on such studies, this investigation focused on the degree of expression and interactions of CRNDE, miR-545-5p and CCND2 within NPC, confirming that CRNDE can promote NPC pathogenesis and development through miR-545-5p/CCND2, consequently providing novel options for NPC therapies.

## Methods

### Sample collection

Globally, 32 frozen NPC biopsies and healthy juxta-positioned tissues were collected from NPC patient cohorts that underwent surgical intervention at The First Affiliated Hospital of Bengbu Medical College. Inclusion criteria consisted of the following: diagnosed as poorly differentiated squamous cell carcinoma by biopsy; no chemoradiotherapy performed prior to enrollment; normal morale and communication. Exclusion criteria consisted of the following: combination of NPC with other malignant tumors; combined heart, liver, kidney and other vital organ lesions; combined pneumonia, hepatitis B and other infectious diseases. All procedures involving human samples in this study were conducted according to the Helsinki declaration and accepted by the Ethics Committee of The First Affiliated Hospital of Bengbu Medical College. Informed consent was collected from all individuals.

### Cell culture and transfection

*H. sapiens* NPC HNE-1, CNE-2Z, 5-8F and healthy nasopharyngeal epithelial cell line NP69 were from XiangYa School of Medicine of Central South University [Hunan, China]. All NPC cell lines were grown within RPMI-1640 medium [Gibco™, USA], augmented by 10% fetal bovine serum [FBS, Gibco™, USA], and incubated at 37 °C/5% CO_2_. NP69 cell cultures were grown within keratinocyte serum-free medium [Gibco™, USA]. Small interfering RNA against CRNDE (si-CRNDE) and relevant controls (si-NC), miR-497-5p mimics and relevant controls (miR-NC), together with miR-497-5p inhibitor and relevant controls (miR-inhibitor NC) were all procured from Sangon™ [Shanghai, China]. All transfections employed Lipofectamine 2000® [Thermo™, USA].

### Quantitative reverse-transcription polymerase chain reaction

Total RNA was collected from CNE-2Z/HNE-1 cells using TRIzol® [Ambion™, TX, USA] and reverse transcribed into cDNA through EasyScript® One-step gDNA Removal and cDNA Synthesis SuperMix® kit [TransGen™, China]. Consequently, ABI StepOnePlus® [Bio-Rad™, USA] was utilized for 40 cycles of amplification at 95 °C for 30 s, denaturation at 95 °C for 5 s, and extension at 65 °C for 30 s. GAPDH and U6 served as normalization controls of mRNAs and miRNAs, respectively. The −2ΔCT method was used to calculate relative expression for all investigated miRNAs/transcripts. Primer sequences for RT-qPCR were as follows:

CRNDE (Forward, 5′-GGAAAAATCAAAGTGCTCGAGTGG-3′; Reverse, 5′-TCTTCTGCGTGACAACTGAGGA-3′),

miR-545-5p (Forward, 5′-CGCGCGTCAGTAAATGTTTATT-3′; Reverse, 5′-AGTGCAGGGTCCGAGGTATT-3′),

CCND2 (Forward, 5′-TTTAAGTTTGCCATGTACCCAC-3′; Reverse, 5′-ACGTCTGTGTTGGTGATCTTAG-3′).

### EdU assay

Cells from each group of CNE-2Z and HNE-1, in logarithmic growth phase, were seeded into 6-well plates (1.5 × 10^4^ cells/well). The cells in each group were labeled with EdU detection solution [Beyotime Biotechnology™, China] and consequently fix-treated using 4% paraformaldehyde [Beyotime Biotechnology™, China] at ambient temperature for 15 min. Following wash-steps using PBS, 200 µL/well reaction solution were introduced, and plates placed into incubation for 30 min in darkness at ambient temperature. Following another PBS wash-step, 1 mL Hoechst 33,342 [Beyotime Biotechnology™, China] were added to each well for DNA staining. Following a final PBS wash-step, wells were visualized by laser confocal microscopy.

### CCK-8 assay

HNE-1 cells and CNE-2Z cells, with good growth status post-transfection, were inoculated in 96-well plates (5000 cells/well). Consequently, 10 μL CCK-8 [Beyotime Biotechnology™, China] aliquot were introduced into all wells at 24-, 48-, 72- and 96-h, respectively, and placed into incubation for 120 min. Absorbance (OD) values at 450 nm were measured for individual wells upon a microplate reader, and a proliferation curve was prepared.

### Transwell assay

In the migration experiment, Materigel gel was not paved. In the invasion experiment, Materigel® [Corning™, USA] and Opti-MEM® I Reduced-Serum Medium [Thermo™, USA] were diluted at a ratio of 1: 8, with 50 μL of each chamber spread at the base of the upper-chambers and incubated for 1 h to render it semi-solidified. CNE-2Z and HNE-1 cells were inoculated into the upper-chambers within serum-free RPMI-1640 medium, and 600 μL RPMI-1640 medium carrying 10% FBS were introduced into the lower wells. Regarding the migration experiment, cells were grown for 24 h. Regarding the invasion experiment, cells were grown for 48 h. The upper-chambers were consequently removed, fix-treated using 4% paraformaldehyde for 15 min, dyed using crystal violet for 10 min, subjected to PBS wash-step, followed by careful removal of excess dye using a cotton swab, and finally observed/counted. The cellular population density within five randomly selected fields-of-vision under light microscopy were determined, with all experimental runs performed on three separate occasions.

### Wound-healing assay

Cells were labeled at the bottom of the six-well plate pre-inoculation. At 24 h post-transfection, once cultures obtained 80% confluence, cells were lined—perpendicular to the bottom—with a 100 μL sterile pipettor tip/gun. Linear changes at 0 h and 24 h were observed, and the cellular migrative ability was detected. The calculation used was:

$${\text{Wound healing rate }}\left( \% \right) \, = \, \left[ { \, \left( {0{\text{-h scratch width }}{-}{ 24} {\text{-h scratch width}}} \right) \, / \, 0{\text{-h scratch width }}} \right] \, \times 100 \, \% .$$

### Flow cytometric analyses

In order to assess apoptosis, cultures were collected and resuspended, centrifuged, supernatant discarded, and pre-cooled at 4 °C with D-Hanks [Phygene™, China] (pH = 7.2 ~ 7.4) washing cell precipitation. Consequently, 1 × binding buffer wash-step was performed on culture cells to precipitate once, followed by centrifugation and cell collection. Consequently, 200 μL 1 × binding buffer were used for resuspending cell precipitation. A total of 2 μL Annexin V-APC and PI staining were added, with cultures left at room temperature in darkness for 20–60 min. According to cell volume, 200–300 μL of 1 × binding buffer were added, followed by on-line detection.

In order to detect cell cycle, samples were harvested/fixed overnight using 70% ethanol and pre-cooled at 4 °C. Fixative was then removed and cells washed using PBS at 4 °C once. Cell staining solution was prepared by adding 0.5 mL propidium iodide staining solution to each cell sample, slowly and fully resuspending cell precipitations, followed by 37 °C in darkness for 30 min and temporary storage at 4 °C, or in an ice bath. Following staining, flow cytometry was used to complete detection.

### Dual-luciferase reporter assay

All 3′-UTR CRNDE/CCND2 sequences were amplified using polymerase chain reaction (PCR) and ligated with GV272 vector to construct wild-type CRNDE reporter vector (CRNDE-wt) and mutant-type CRNDE (CRNDE-mut), wild-type CCND2 reporter vector (CCND2-wt) and mutant-type CCND2 (CCND2-mut) luciferase reporter plasmids, respectively. MiR-545-5p mimics, together with corresponding negative control miR-NC, were co-transfected with the two recombinant plasmids into CNE-2Z cells using lipofectamine® 3000 [Invitrogen™, USA]. Luciferase activities for individual study-groups were evaluated in order to analyze possible binding of CRNDE with miR-545-5p, and CCND2 with miR-545-5p.

### Western blotting

Protein content from all tissue/cell line samples were extracted and quantified through BCA kit® [Beyotime Biotechnology™, China]. Following electrophoresis, samples were transferred onto PVDF membranes [Biosharp™, China], milk-blocked and consequently hybridized with a variety of primary antibodies [1:1000 dilution, Proteintech Group™, China] and incubated overnight (4 °C). This was followed by placing into incubation with secondary antibodies [1:1000 dilution, Proteintech Group™, China] at room temperature, membrane scanning and eventual observation of band shifts.

### Xenograft tumor model

BALB/c nude murines (female) were segregated into two cohort-groups, sh-CRNDE NC (sh-NC) and sh-CRNDE. The tumor cells of each experimental group in the logarithmic growth phase were counted by blood cell counting plate, and finally suspended with the required volume of D-Hanks or PBS. Following preparation of tumor cells (5.00 E+06 cells/murine), a disposable sterile syringe was utilized to aspirate cells and consequently inject 200 µL into each nude murine. At 24-days following subcutaneous injection, all murines were euthanized using 2% pentobarbital sodium (0.5 mL), followed by cervical-dislocation sacrifice. Selected tumor samples were fix-treated with 4% paraformaldehyde for immunohistochemical analyses. All other samples were kept at − 80 °C for Western blot assays.

### Immunohistochemistry

The transplanted tumor was fixed with formalin for 48 h, soaked in 4% paraffin and sliced. Three xylene washing cycles were performed every 15 min, followed by three washing cycles using a differing ethanol concentration in each wash cycle (100%/95%/-80%) every 5 min. Consequently, high-pressure antigen repair was performed, using a high-pressure cooker containing 2 L of double-distilled water treated with 40 mL of pH 8.0 EDTA repair solution. This solution was consequently heated to boiling point. Following from this step, tumor slices (together with the dyeing frame) were placed into the repair solution for two minutes in order to allow boiling repair. The procedure was then halted, and the solution was allowed to cool by adding a moderate volume of distilled water. Tumor slices were consequently extracted individually. Tissue-regions from each tumor slice were marked using an immunohistochemical pen and placed in an incubator. In addition, 3% hydrogen peroxide [Zsbio™, China] was added to tissue samples, followed by incubation at room temperature for 20 min. Following sample segment washing, drying, Ki67-treatment [Zsbio™, China] and 37 °C incubation for 60 min, a secondary antibody [Zsbio™, China] was added, followed by incubation at 37 °C for 20 min. Following further wash/dry-steps, DAB coloring agent [Zsbio™, China] was added, with monitoring of color time under a microscope for any positive staining stop-colorations. Once coloration step was stopped, samples were treated with hematoxylin [Baso™, China] staining for one minute, followed by 1% hydrochloric acid treatment (a few seconds). Finally, samples were dehydrated, rendered transparent, and sealed for image capturing.

### Statistical analysis

SPSS21.0® software was employed for analyzing experimental datasets, represented as mean ± standard deviation [SPSS, Inc™, USA]. One-/two-way variance analyses were employed for comparative analyses. LSD-t test was employed for group comparisons. A p-value of < 0.05 conferred statistical significance.

## Results

### Down-regulation of CRNDE inhibits NPC proliferative, migrative and invasive properties

**Fig. 1:**
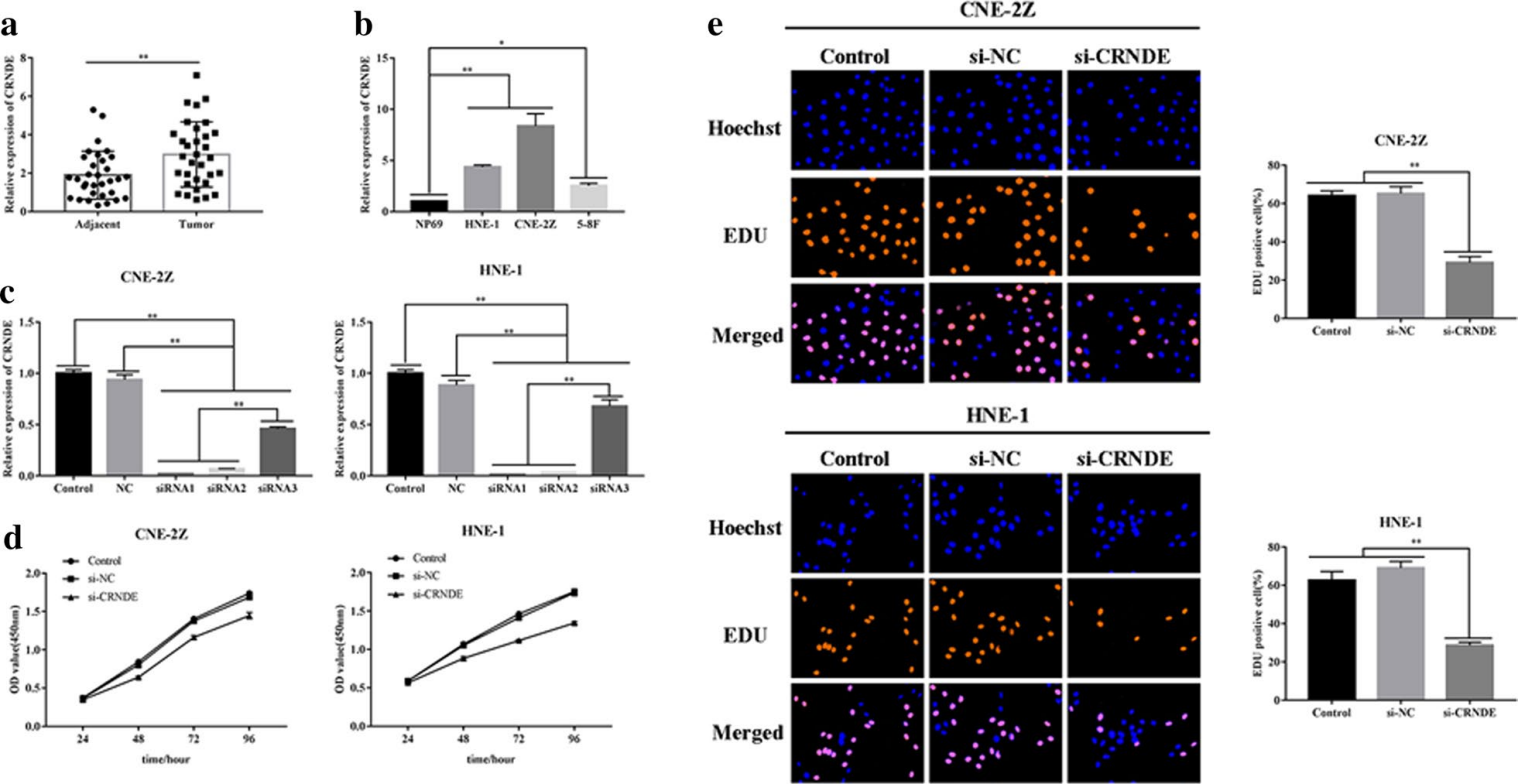
CRNDE knockdown blocks NPC proliferation. **a** CRNDE was expressed in NPC adjacent tissue samples. **b** CRNDE level within NP69, CNE-2Z, HNE-1, 5-8F cellular lineages. **c** Relative expression of CRNDE in NPC cells (CNE-2Z, HNE-1) following transfection of siRNAs (siRNA1, siRNA2, siRNA3) and negative control (si-NC). **d** CCK-8 assays analyzed NPC proliferative property (CNE-2Z, HNE-1). **e** The EdU assays analyzed NPC proliferative property (CNE-2Z, HNE-1) (×200). *p < 0.05; **p < 0.01

RT-qPCR was employed for gene expression analysis of CRNDE within 32 NPC and matching adjacent tissues. CRNDE expression in NPC tissues was found to be highly up-regulated in comparison to adjacent tissue levels (p < 0.01) (Fig. 1a). In comparison to healthy nasopharyngeal epithelial cells (NP69), up-regulation was observed for CRNDE in NPC cell lines CNE-2Z, HNE-1 (P < 0.01) and 5-8F (P < 0.05) (Fig. 1b). Consequently, siRNAs (siRNA1, siRNA2 and siRNA3) were used for inhibiting CRNDE expression within CNE-2Z/HNE-1 cell lines. According to the best inhibitory effect, siRNA1 was selected for subsequent experiments (P < 0.01) (Fig. 1c).

**Fig. 2:**
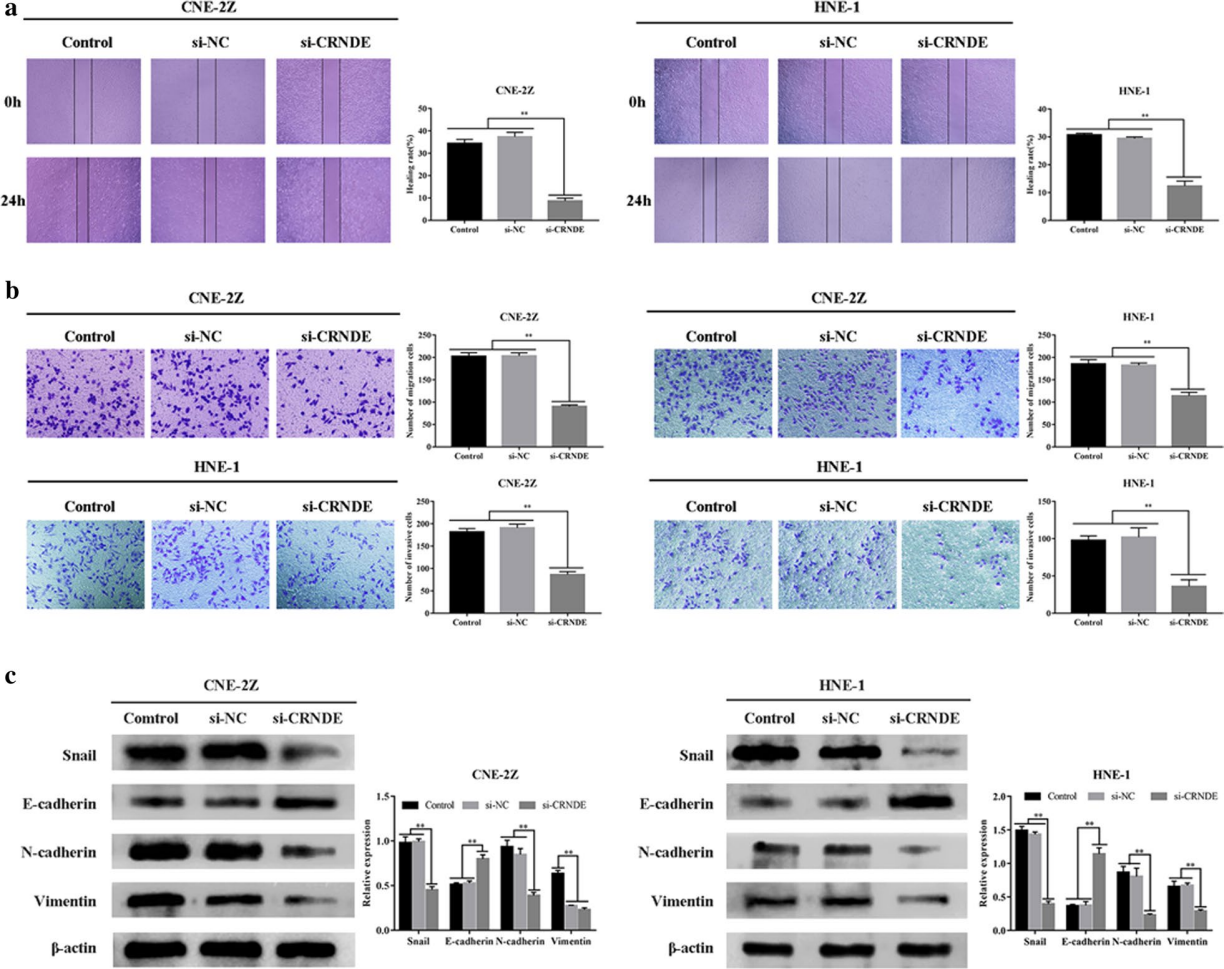
Down-regulated CRNDE reduced NPC cell line metastases (CNE-2Z, HNE-1). **a** Wound healing assays detected down-regulated CRNDE inhibiting NPC migrative property (CNE-2Z, HNE-1) (×40). **b** CRNDE knockdown reduced migration/invasion of NPC cells (CNE-2Z, HNE-1) as detected by Transwell assay (×200). **c** Western blot analyzed proteomic content for EMT-related proteins in NPC (CNE2Z, HNE-1) when CRNDE was down-regulated. *p < 0.05; **p < 0.01

CCK-8 assays indicated that CRNDE down-regulation induced drastic regulatory influence on cellular viability in the NPC group, in comparison to the control group and NC (P < 0.01) (Fig. 1d). EdU assays revealed that CRNDE knockdown could significantly reduce the number of positive cells in CNE-2Z and HNE-1 cell lines (P < 0.01) (Fig. 1e). In addition, transwell/wound-healing assays demonstrated that CRNDE silencing in CNE-2Z and HNE-1 cell lines inhibited their migrative/ and invasive properties (P < 0.01) (Fig. 2a, b).

EMT-linked proteins Snail, E-cadherin, N-cadherin and Vimentin were identified via Western blotting. In comparison to the control group and NC, E-cadherin levels within the si-CRNDE group were elevated, with down-regulated Snail, N-cadherin and Vimentin levels (P < 0.01) (Fig. 2c). Such findings suggest CRNDE to exacerbate NPC progression.

### Down-regulation of CRNDE inhibits apoptosis of NPC cells and affects cell cycle

**Fig. 3:**
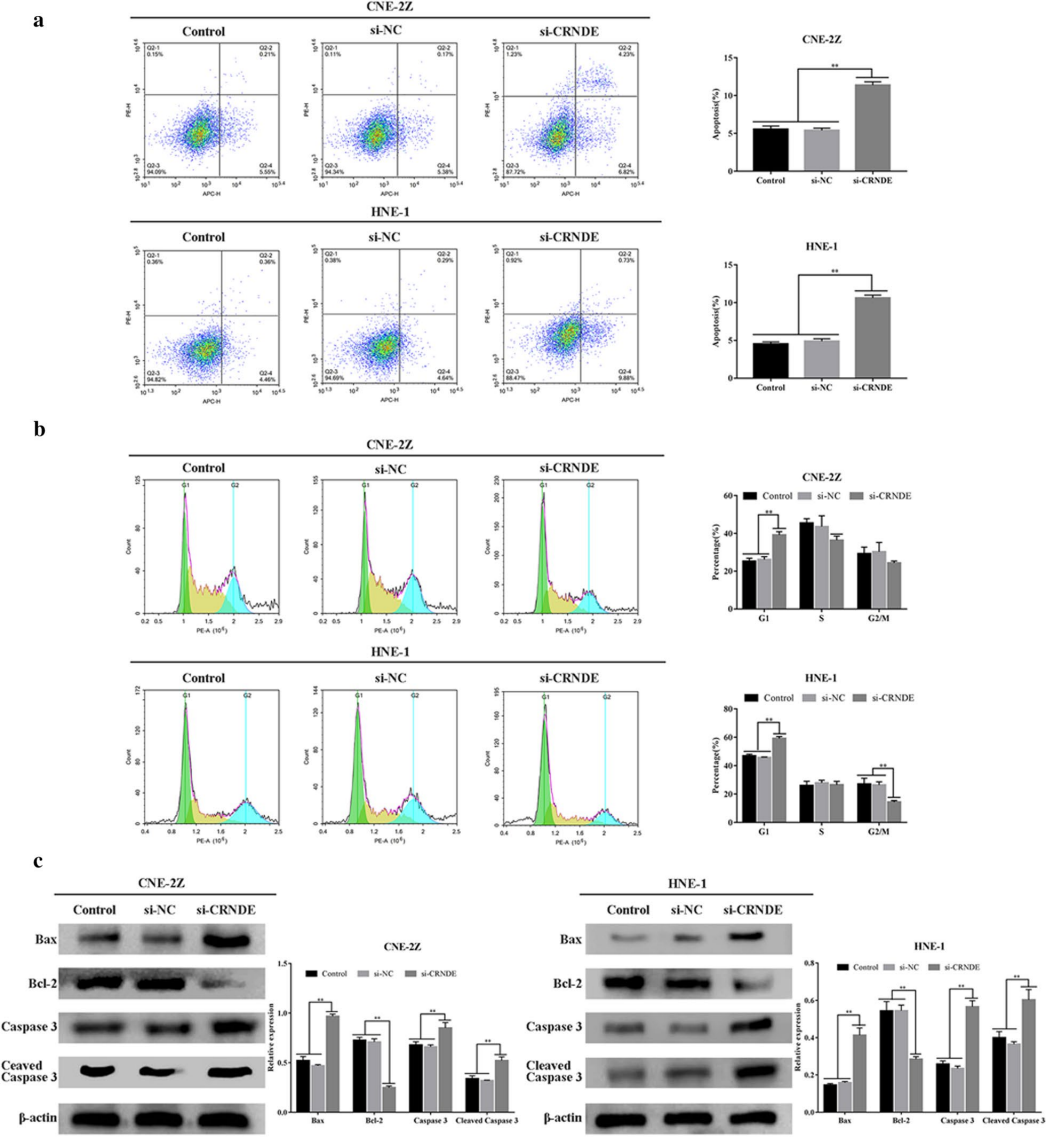
Reduced CRNDE level enhanced apoptotic rate and influenced NPC cellular cycle progression of (CNE2Z, HNE-1). **a** Flow cytometry demonstrated that the apoptotic activity of NPC cell lines (CNE2Z, HNE-1) was enhanced following the down-regulation of CRNDE. **b** The changes of cell cycle progression of NPC cells(CNE2Z, HNE-1) induced by down-regulation of CRNDE, as detected by flow cytometry.** c** Western blot analyzed proteomic content for apoptosis-related proteins in NPC cellular lineages (CNE2Z, HNE-1) when CRNDE was down-regulated. *p < 0.05; **p < 0.01

CRNDE influences on NPC pathology were studied by detecting apoptotic activities and NPC cell cycle status. Flow cytometry demonstrated that apoptotic rates in the si-CRNDE group were elevated, in comparison to control/NC groups, and the si-CRNDE groups of CNE-2Z/HNE-1 cell lines exhibited G0/G1 phase arrest (P < 0.01) (Fig. 3a, b).

Apoptosis-related proteins were identified through Western blotting. Suppression of CRNDE promoted Bax/Cleaved Caspase 3 expression, and down-regulated Bcl-2 (P < 0.01) (Fig. 3c). Such experimental results highlighted knocking-down of CRNDE to enhance NPC apoptotic activity via mitochondrial apoptosis-related pathway triggering.

### CRNDE has a targeting relationship with miR-545-5p

**Fig. 4:**
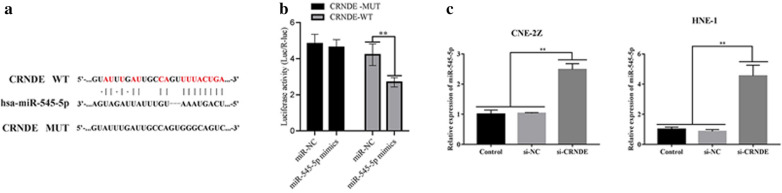
CRNDE sponges miR-545-5p within NPC. **a** Prediction of targeted binding sites (CRNDE/miR-545-5p) by Tiana Tool. **b** Following co-transfection of miR-545-5p or miR-NC, the luciferase function in CRNDE-WT/CRNDE-MUT in CNE-2Z were detected. **c** The effect of si-CRNDE transfection over miR-545-5p expression in NPC (CNE2Z, HNE-1). *p < 0.05; **p < 0.01

Tiana Tool (v.2) was used to predict any downstream regulatory correlation for miR-545-5p/CRNDE. The results highlighted that CRNDE possesses a binding site for miR-545-5p (Fig. 4a). Firstly, it was confirmed by dual-luciferase reporter assay that over-expressing miR-545-5p blocked luciferase function within CRNDE-wt. This indicated that miR-545-5p binds onto CRNDE through sequence-specific manners (P < 0.01) (Fig. 4b). Furthermore, RT-qPCR was employed for analyzing relative expression levels of miR-545-5p within the control, si-NC, and si-CRNDE groups of CNE-2Z/HNE-1 cell lines. The assay outcomes demonstrated that miR-545-5p was up-regulated within the si-CRNDE group, in comparison to control/si-NC groups (P < 0.01) (Fig. 4c). According to such outcomes, CRNDE can target and successfully downregulate miR-545-5p.

### miR-545-5p regulates NPC proliferative, migrative and invasive properties

RT-qPCR was employed for analyzing miR-545-5p expression within NP69, CNE-2Z, HNE-1 and 5-8F. Consequently, miR-545-5p was down-regulated within CNE-2Z, HNE-1 and 5-8F, in view of healthy nasopharyngeal NP69 cell line (P < 0.01). Since miR-545-5p levels within CNE-2Z and HNE-1 lineages were relatively low, these two cell lines were selected for subsequent experiments.

**Fig. 5:**
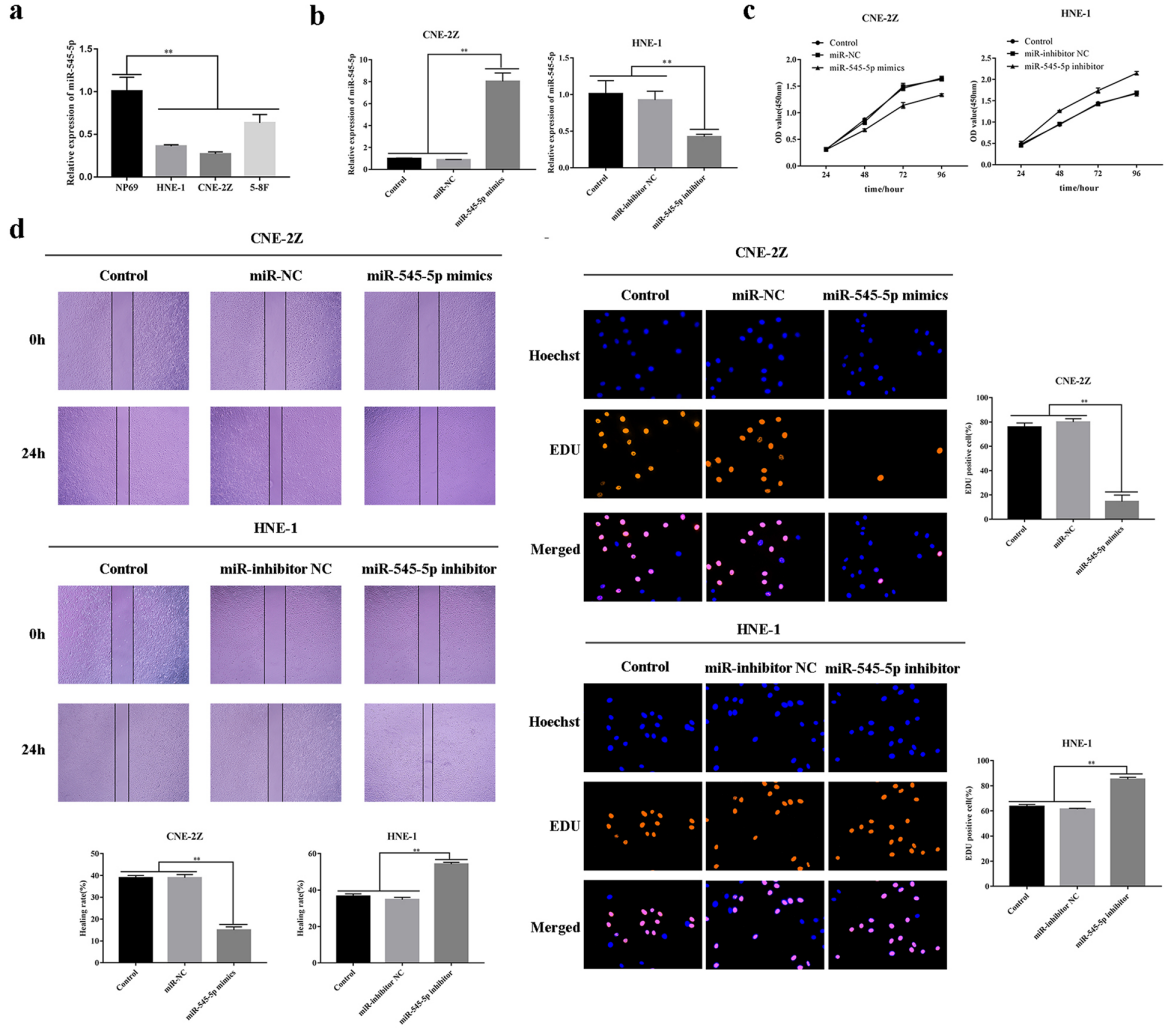
Up-regulation/down-regulation influences by miR-545-5p on NPC proliferative/migrative properties. **a** miR-545-5p expression within multiple NPC cellular lineages (CNE-2Z, HNE-1, 5-8F). **b** miR-545-5p expression within NPC (CNE-2Z, HNE-1) following transfection of miR-545-5p mimics/inhibitor. **c** CCK-8 assays analyzed NPC proliferative property (CNE-2Z, HNE-1). **d** The relationship between the expression of miR-545-5p and the migration of NPC cells (CNE-2Z, HNE-1) was studied by wound healing assay (×40). **e** The proliferation of NPC cells was analyzed by the EdU assay (CNE2Z, HNE-1) (×200) *p < 0.05; **p < 0.01

**Fig. 6:**
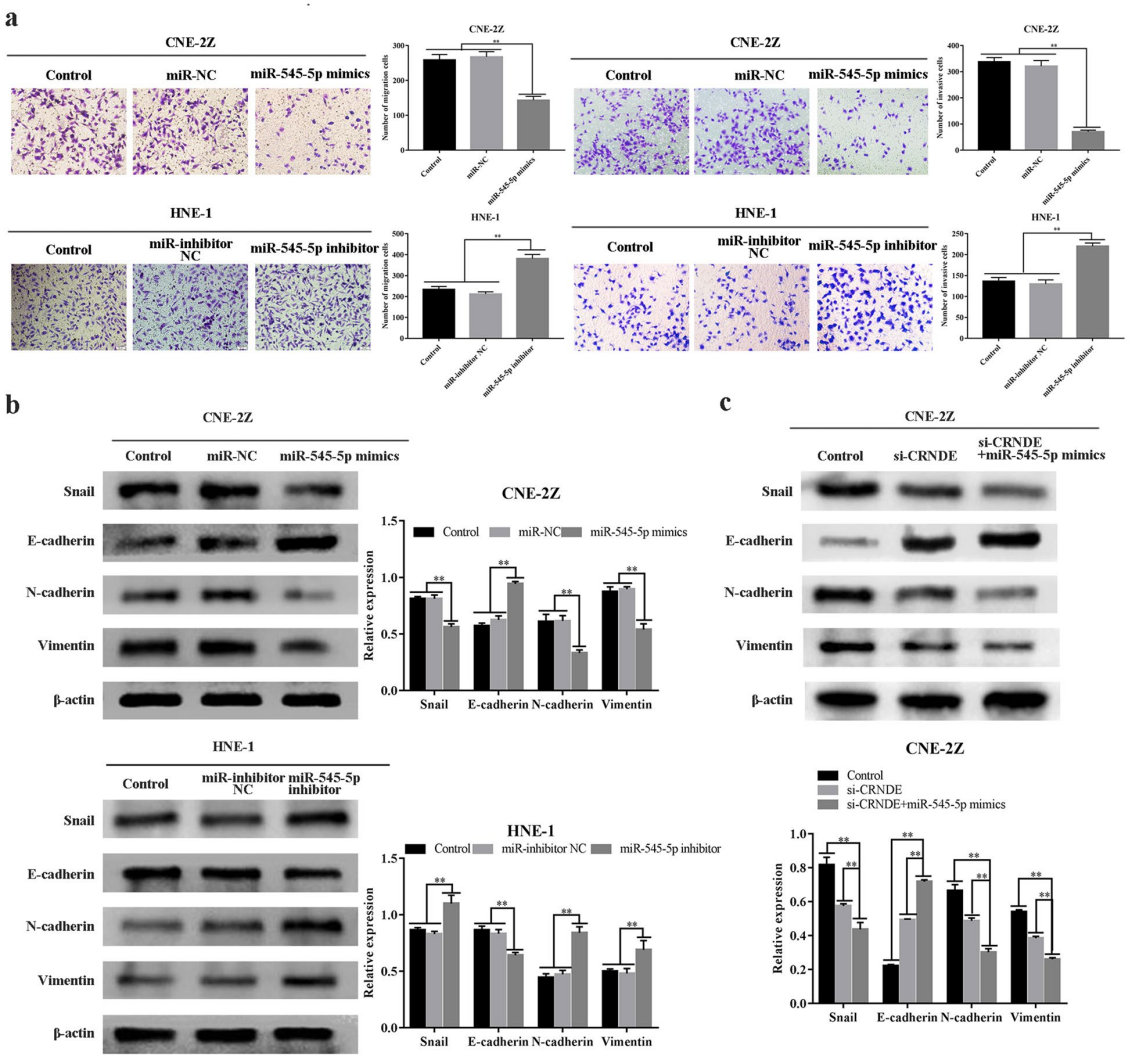
Effects of up-regulation/down-regulation of miR-545-5p upon invasion and migration of NPC cell lines. **a** The effect of miR-545-5p on migration and invasion of NPC cells (CNE-2Z, HNE-1) was detected by transwell assay (×200). **b** Western blot was used to detect the effect of miR-545-5p mimics or miR-545-5p inhibitor transfection on the expression of EMT-related proteins in NPC cells (CNE-2Z, HNE-1). **c** Western blot was used to detect the expression of EMT-related proteins when si-CRNDE was down-regulated and miR-545-5p mimics were co-transfected. *p < 0.05; **p < 0.01

CNE-2Z cultures were subjected to miR-545-5p mimics-transfection, while HNE-1 underwent miR-545-5p inhibitor transfection (P < 0.01) (Fig. 5b). CCK-8 assays demonstrated that, in comparison to the control group and NC, the cellular proliferative property was reduced following miR-545-5p up-regulation, though increased following miR-545-5p down-regulation (P < 0.01) (Fig. 6c).

EdU assays indicated that miR-545-5p up-regulation significantly reduced cellular proliferative property, while miR-545-5p down-regulation had opposing effects (P < 0.01) (Fig. 5e). In addition, transwell assays and wound-healing assays demonstrated that the migrative and invasive properties of CNE-2Z/HNE-1 cell lines were inhibited post-miR-545-5p up-regulation, with the latter’s down-regulation having opposing effects (P < 0.01) (Figs. 5d, 6a).

Snail, E-cadherin, N-cadherin and Vimentin were analyzed through Western blotting. E-cadherin level within the miR-545-5p mimic-group was higher in comparison to the control/NC groups, while Snail, N-cadherin and Vimentin expression was reduced (P < 0.01). Proteomic expression profile was reversed post-miR-545-5p (P < 0.01) (Fig. 6b). Overexpression of miR-545-5p, combined with CRNDE down-regulation promotes si-CRNDE-mediated inhibition of cell migrative and invasive properties (P < 0.01) (Fig. 6c). Such outcomes suggest that miR-545-5p can affect NPC development.

### miR-545-5p influences on apoptosis and cell cycle during NPC progression

**Fig. 7:**
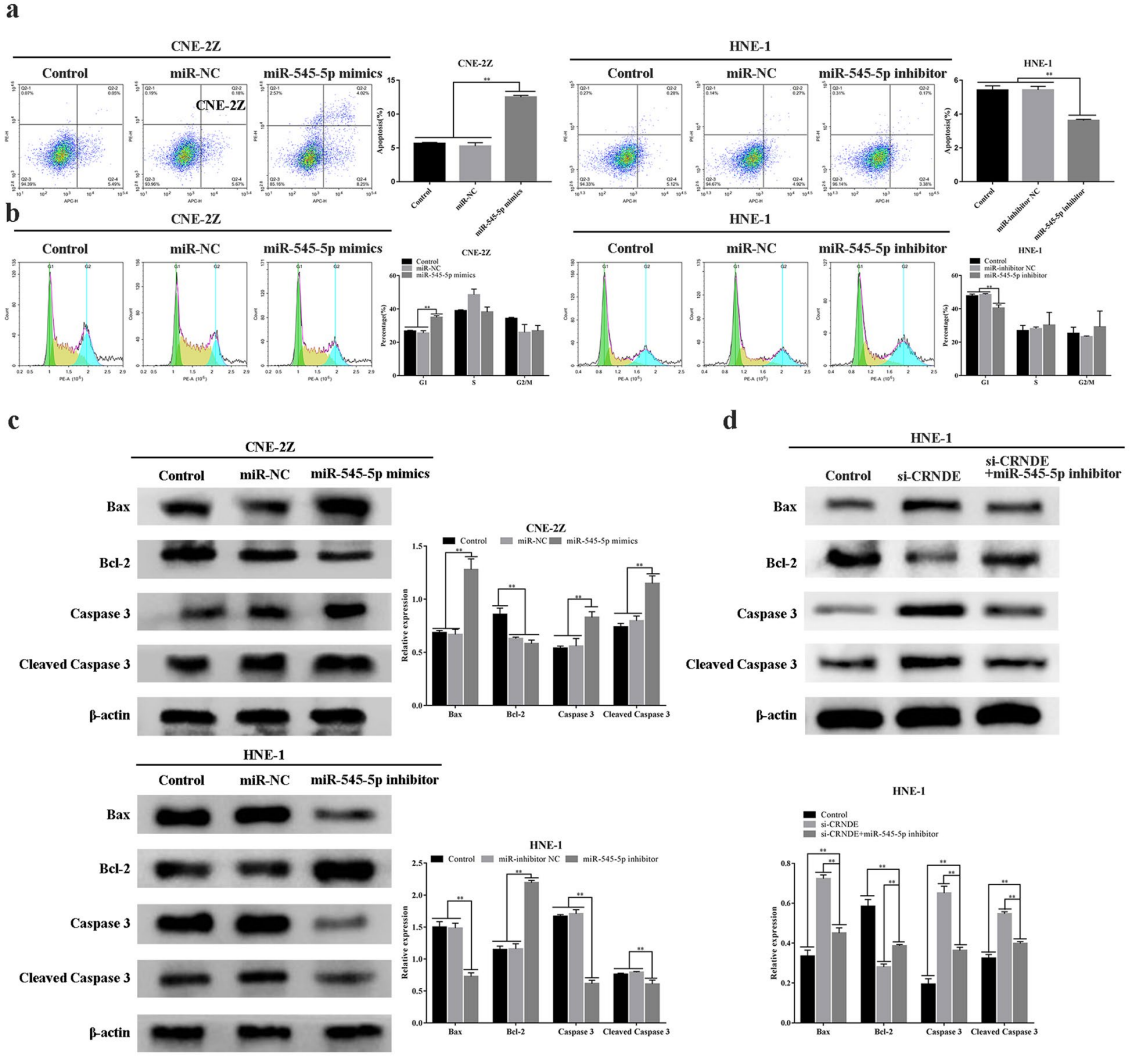
Effect of miR-545-5p on apoptosis and cell cycle progression of NPC cells. **a** Flow cytometry was used to detect the effect of miR-545-5p on apoptosis of NPC cells (CNE-2Z, HNE-1). **b** The effect of miR-545-5p expression on the cell cycle progression of NPC cells (CNE-2Z, HNE-1) was detected by flow cytometry. **c** Western blot was used to detect the expression of apoptosis-related proteins when miR-545-5p was overexpressed or knocked down. **d** The expression of apoptosis-related proteins was detected by Western blot following co-transfection of si-CRNDE and miR-545-5p inhibitor. *p < 0.05; **p < 0.01

Similar to CRNDE, miR-545-5p influences upon NPC pathology were studied by detecting the apoptotic activity and cell cycle status of NPC cells. Flow cytometry revealed that, in comparison to the corresponding control/NC groups, the apoptotic rate of miR-545-5p-mimics-group in CNE-2Z was increased (P < 0.01), together with an increased degree of cells within G1 phase (P < 0.01). The apoptotic rate of miR-545-5p-inhibitor-group in HNE-1 cell lines was decreased (P < 0.01), with cellular degree in G1 phase significantly decreased as well (P < 0.01) (Fig. 7a, b). Western blotting demonstrated that post-miR-545-5p up-regulation of Bax and Cleaved Caspase 3 proteins were up-regulated, with concomitant Bcl-2 down-regulation. Conversely, miR-545-5p down-regulation had opposing function (P < 0.01) (Fig. 7c). Regarding CRNDE down-regulation, miR-545-5p down-regulation reduced inhibitory functions imposed by si-CRNDE on cellular proliferative property (P < 0.01) (Fig. 7d). Such outcomes indicated that miR-545-5p could affect NPC cellular cycle to promote apoptosis.

### CCND2 is targeted by miR-545-5p and CRNDE acts as ceRNA, regulating CCND2 expression by miR-545-5p

**Fig. 8:**
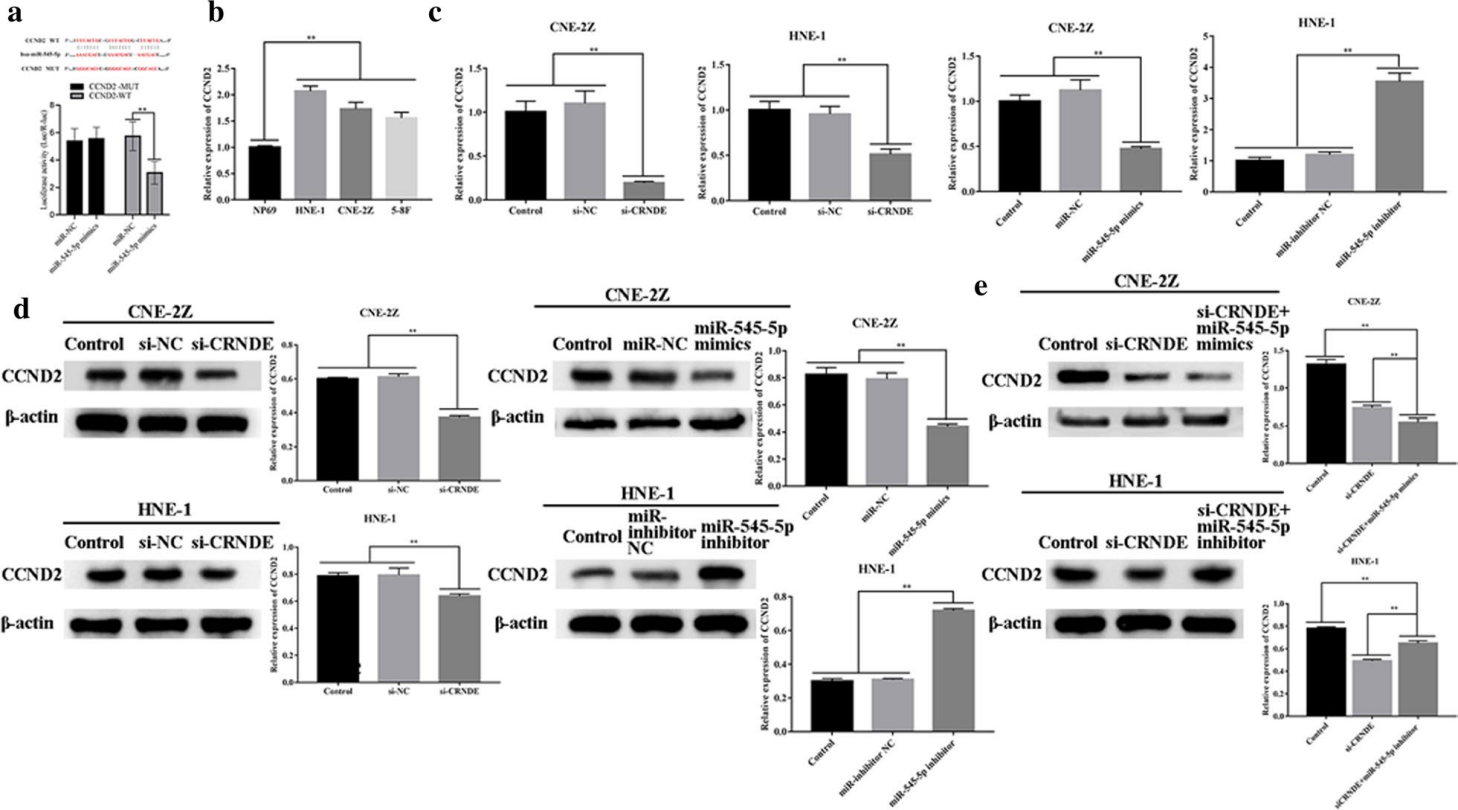
CCND2 expression was modulated by the CRNDE/miR-545-5p axis. **a** The prediction of TargetScan and the detection of dual luciferase reporter assays demonstrated that CCND2 was a target of miR-545-5p. **b** CCND2 was expressed in a human normal nasopharyngeal epithelial cell line (NP69) and NPC cell lines (CNE-2Z, HNE-1, 5-8F). **c** Downregulating CRNDE or upregulating miR-545-5p can affect expression of CCND2 in NPC cells (CNE-2Z, HNE-1). **d** Western blot was used to detect the changes of CCND2 expression in NPC cells (CNE-2Z, HNE-1) when miR-545-5p was up-regulated or down-regulated. **e** Western blot was used to detect the expression of CCND2 when CRNDE was down-regulated and miR-545-5p was up-regulated or down-regulated. *p < 0.05; **p < 0.01

Targetscan predicted that CCND2 can be a potential target of miR-545-5p. CRNDE and CCND2 have identical binding sequences as miR-545-5p, suggesting that CRNDE and CCND2 could compete to bind miR-545-5p (Fig. 8a).The dual-luciferase reporter assay confirmed this, indicating that miR-545-5p over-expression thwarted luciferase function within CCND2-wt (P < 0.01) (Fig. 8a). RT-qPCR demonstrated miR-545-5p up-regulation within CNE-2Z, HNE-1 and 5-8F in comparison with NP69 (P < 0.01) (Fig. 8b). Furthermore, CCND2 down-regulation was noted post-CRNDE down-regulation or post-miR-545-5p up-regulation. Such results gave opposing trends upon miR-545-5p down-regulation (P < 0.01) (Fig. 8c). Western blot outcomes also demonstrated CCND2 down-regulation post-CRNDE down-regulation or miR-545-5p up-regulation, with such influences being rescued post-miR-545-5p inhibitor treatment (P < 0.01) (Fig. 8d).

Furthermore, stemming from CRNDE down-regulation, concomitant miR-545-5p down-regulation weakens inhibitory influence by CRNDE down-regulation, upon CCND2 expression (P < 0.01) (Fig. 8e). In essence, CRNDE is a ceRNA, whereby miR-545-5p regulates CCND2 expression.

### Knockdown of CRNDE inhibits xenograft growth associated with miR-545-5p/CCND2 axis

**Fig. 9:**
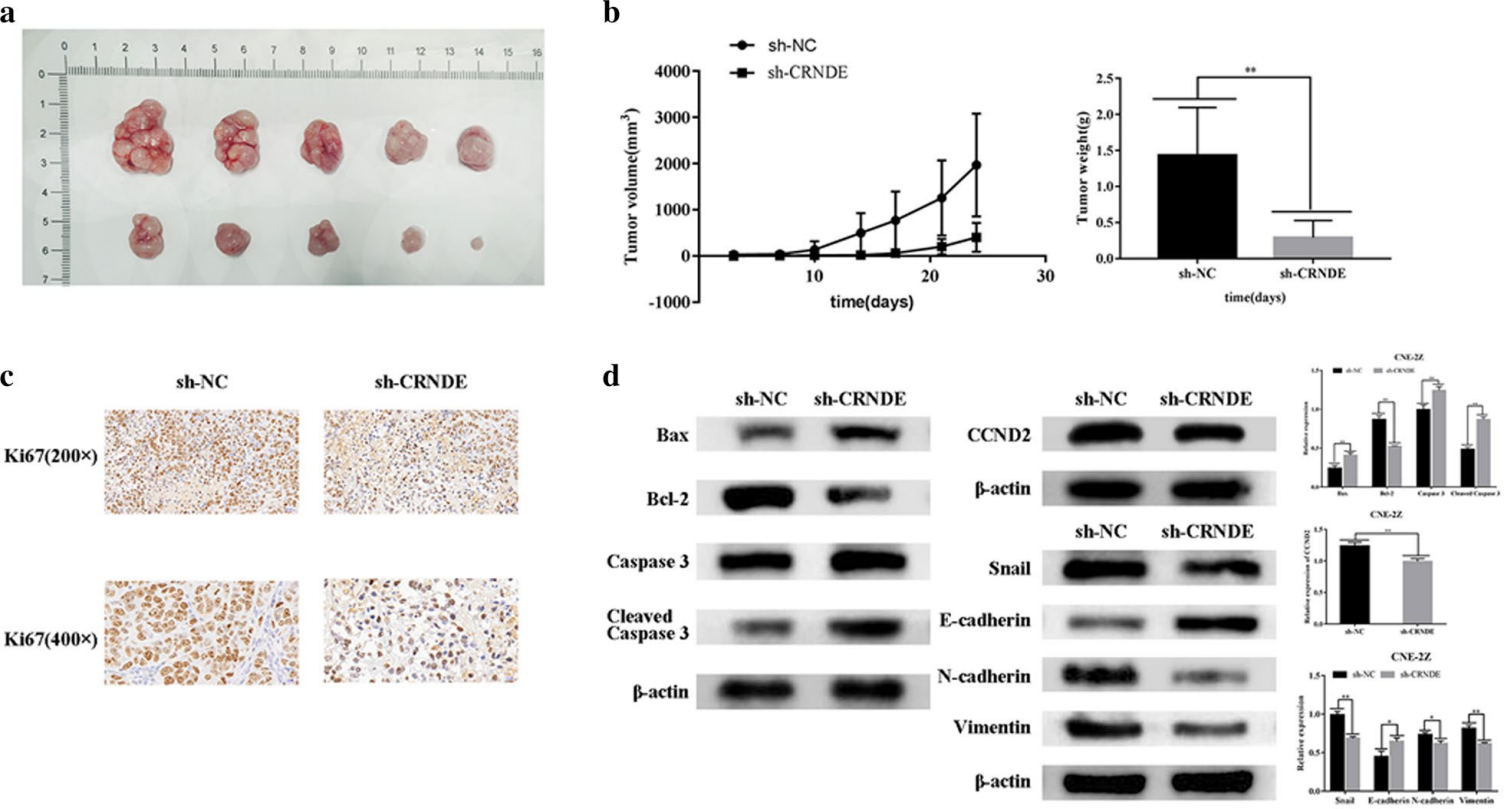
CRNDE silencing hinders tumour growth in vivo. **a** Xenograft tumours were removed from sh-CRNDE or sh-NC groups. **b** Tumor volume and weight in the sh-CRNDE or sh-NC groups were measured and calculated. **c** Immunohistochemical staining for Ki-67 in xenografts. **d** Western blotting was used to detect the expression of EMT-related proteins, apoptosis-related proteins and CCND2 protein in each group. *p < 0.05; **p < 0.01

In order to study the effect of CRNDE knockdown in vivo, a murine xenograft model was developed via injection of sh-CRNDE or its related negative control (sh-NC) (Fig. 9a). In comparison to the sh-NC group, tumor volume/weight in sh-CRNDE group was decreased (P < 0.01) (Fig. 9b). Immunohistochemistry demonstrated that the brown color of sh-CRNDE group was significantly reduced in comparison to NC group, post-Ki67 staining and DAB coloring (Fig. 9c). Western blotting demonstrated down-regulation of CCND2 within sh-CRNDE group, together with up-regulation of Bax/Cleaved Caspase 3. Bcl-2 was also down-regulated. In addition, E-cadherin was up-regulated while Snail, N-cadherin and Vimentin were down-regulated (P < 0.01) (Fig. 9d). It was further confirmed that knockdown of CRNDE inhibits the expression of CCND2 and inhibits the progression and metastasis of NPC in vivo.

## Discussion

NPC is a highly prevalent head and neck malignant tumor with significant regionality [19]. Investigation of the pathogenesis and development mechanism/s of NPC can open a broader idea for the treatment of NPC. Following the above-described experiments, our investigation found that in NPC cells, miR-545-5p was down-regulated, together with CRNDE and CCND2 up-regulation. CRNDE can upregulate CCND2 through sponge-binding of miR-545-5p, affecting NPC proliferation, migration, invasion and apoptotic activities.

LncRNAs represent a non-protein-coding RNA molecular class which have pivotal parts within tumor development, treatment and prognosis. In recent years, CRNDE has been confirmed as up-regulated within colon cancer and has promoted the development of other tumors. Notwithstanding, CRNDE roles within the process of NPC is rarely reported. This study demonstrated that CRNDE was up-regulated within carcinoma tissues of NPC patients. In comparison to normal nasopharyngeal epithelial cells, CRNDE was up-regulated within NPC lineages and promotes NPC proliferative, migrative and invasive properties, together with inhibiting apoptosis. This ability to promote cancer was suppressed when CRNDE was knocked down. In mitochondrial apoptosis-related pathways, anti-apoptotic gene Bcl-2 and pro-apoptotic Bax are two key influencing genomic factors [20]. Caspases are essential components of the apoptotic mechanism, especially caspase-3 [21]. Following knock-down of CRNDE, Bcl-2 was drastically down-regulated, with concomitant up-regulation of Bax and caspase-3. Epithelial-mesenchymal transition (EMT) describes the gradual event of epithelial cells obtaining mesenchymal profiles, with consequently serious contributions to cancer progression, metastasis and drug resistance [22, 23]. Snail can endow tumors with stem cell-type characteristics and exacerbate chemoresistance, patient relapses and EMT [24]. The reduction of E-cadherin, cytokeratin cytoskeleton replacement by Vimentin, together with mesenchymal cell morphological manifestations, are all considered to be EMT characteristics. E-cadherin increase, coupled with Vimentin reductions, can partially regulate NPC metastasis and invasiveness. Therefore, we further studied the influence of CRNDE on EMT in NPC cell lines. This investigation demonstrated that post-CRNDE knock-down, E-cadherin was drastically down-regulated, with concomitant up-regulation of N-cadherin and Vimentin. In essence, such outcomes indicate that CRNDE accelerates NPC progression.

The competitive endogenous RNA (ceRNA) hypothesis of lncRNAs shows that when miRNA response elements (MRE) bind to miRNA, miRNA-regulated genes can be silenced [25]. Based on this, we launched a series of studies on CRNDE and ceRNA regulatory networks. Bioinformatics-based approaches were used for predicting CRNDE and miR-545-5p downstream targeting interplays, and ultimately postulating that CRNDE can affect NPC development through miR-545-5p modulation—as confirmed by dual-luciferase reporter assay. Consequently, this study describes the first report on miR-545-5p overexpression leading to thwarting of the proliferative, migrative and invasive properties of NPC, together with enhanced apoptotic activity. When miR-545-5p was inhibited, the opposite results were obtained. This study thus revealed that miR-545-5p is intimately linked to NPC tumorigenesis and development.

CCND2 is a cyclin D family protein, and its kinase activity promotes tumorigenesis by enhancing signal transduction, mediated by cyclin-dependent kinase (Cdk) [26]. Bioinformatics predicted CCND2 was a downstream target for miR-545-5p, and we further verified whether CRNDE regulated CCND2 as a ceRNA. This study demonstrated that CCND2 down-regulation occurred following CRNDE down-regulation, with this influence rescued by anti-miR-545-5p. Once miR-545-5p is overexpressed, it will form a synergistic effect with CRNDE knockdown. Therefore, CRNDE can regulate tumorigenesis and development of NPC by serving as ceRNA of CCND2. Nevertheless, additional in vivo studies are required to further validate such findings. In addition, more detailed mechanisms for CRNDE regarding NPC tumorigenesis and development must be explored further.

## Conclusion

This study identified that CRNDE, as an oncogene for NPC, was highly expressed within NPC biopsies and cellular lineages. In addition, CRNDE and CCND2 have binding sites with miR-545-5p. CRNDE knockdown can inhibit CCND2 within NPC, and this phenomenon is reversed when miR-545-5p is down-regulated. Therefore, CRNDE acts as a ceRNA for sponging miR-545-5p, resulting in up-regulation of CCND2 expression. In summary, CRNDE/miR-545-5p both have potential application value in the treatment of NPC, which can be employed as novel tumor biomarkers for NPC and provide new strategies for early NPC diagnosis and its targeted therapy.

## Data Availability

All the experimental procedures were approved and executed in accordance with the first affiliated hospital of Bengbu Medical College.

## Abbreviations

LncRNAs: Long non-coding RNAs
NPC: Nasopharyngeal carcinoma
miRNAs: MicroRNAs
CCND2: Cyclin D2
EMT: Epithelial-mesenchymal transition
RT-qPCR: Quantitative real-time polymerase chain reaction
MRE: MiRNA response elements
ceRNA: The competitive endogenous RNA
